# The effect of microvesicles derived from K562 cells on proliferation and apoptosis of human bone marrow mesenchymal stem cells

**DOI:** 10.22038/IJBMS.2023.66903.14675

**Published:** 2023-03

**Authors:** Neda Rassaei, Mahnoosh Abbaszade Dibavar, Masoud Soleimani, Amir Atashi, Mohammad Hossein Mohammadi, Mehdi Allahbakhshian Farsani, Shaghayegh Shahsavan

**Affiliations:** 1 Department of Hematology, Faculty of Medical Sciences, Tarbiat Modares University,Tehran, Iran; 2 Department of Hematology and Blood Banking, School of Allied Medical Sciences, Shahid Beheshti University of Medical Sciences, Tehran, Iran; 3 Stem Cell and Tissue Engineering Research Center, Shahroud University of Medical Sciences, Shahroud, Iran; 4 HSCT Research Center, Laboratory Hematology and Blood Banking Department, School of Allied Medical Sciences, Shahid Beheshti University of Medical Sciences, Tehran, Iran; 5 HSCT Research Center, Shahid Beheshti University of Medical Sciences, Tehran, Iran

**Keywords:** Apoptosis, K562 cell line, Mesenchymal stem cell, Microvesicles, Survival

## Abstract

**Objective(s):**

Microvesicles (MVs) are small membrane-bound particles that act as a vehicle to transfer their contents, such as proteins, RNAs, and miRNAs, to the target cells, making them undergo several changes. Depending on the origin and the target cell, MVs may cause cell survival or apoptosis. This study investigated the effects of MVs released from the leukemic K562 cell line on the human bone marrow mesenchymal stem cells (hBM-MSCs) to evaluate changes in the survival or apoptosis of the cells in an *in vitro* system.

**Materials and Methods:**

In this experimental study, we added the isolated MVs from the K562 cell line to hBM-MSCs, and after three and then seven days, subsequently cell count, cell viability, transmission electron microscopy, tracing MVs by carboxyfluorescein diacetate, succinimidyl ester (CFSE) solution, flow cytometry analysis for Annexin-V/PI staining and qPCR for the evaluation of *BCL-*2, *KI67*, and *BAX* expression were carried out. On the 10^th^ day of the culture, hBM-MSCs were examined by Oil red O and Alizarin Red staining to evaluate their differentiation into adipocytes and osteoblasts.

**Results:**

There was a significant decrease in cell viability and *KI67* and *BCL-2* expression; however, *BAX* was significantly upregulated in the hBM-MSCs compared to control groups. Annexin-V/PI staining results also showed the apoptotic effects of K562-MVs on hBM-MSCs. Moreover, the differentiation of hBM-MSCs into adipocytes and osteoblasts was not observed.

**Conclusion:**

MVs from the leukemic cell line could affect the viability of normal hBM-MSCs and induce cell apoptosis.

## Introduction

Chronic myeloid leukemia (CML) is a myelodysplastic neoplasm that accounts for about 15% of all cases of leukemia in adults (1, 2). CML is characterized by the Philadelphia chromosome, which results from chromosomal translocation t(9;22)(q34;q11) and fusion of the *BCR *and *ABL* genes to generate the BCR-ABL protein (3, 4). The constitutive tyrosine kinase activity of this protein causes the malignant transformation of hematopoietic cells by altering cell proliferation, differentiation, and survival (5). 

CML is a disease that originates in the bone marrow microenvironment. Hematopoietic stem cells (HSCs), leukemic stem cells, and mesenchymal stem cells (MSCs) are located in the bone marrow niche. MSCs are multipotent cells in the bone marrow stroma, providing a supportive micro-environment for the differentiation of HSCs into functional adult cells. Hence they play an important role in the pathogenesis of hematological disorders (6, 7). In order to provide an appropriate microenvironment, various cells in the bone marrow should interact with each other. There are several ways for the cells to communicate with each other, such as the secretion of cytokines and cell-cell interaction. However, one of the considerable approaches has recently been the production of extracellular vesicles released by the cells. Tumor cells can release lots of microvesicles (MVs) by outer cell membrane budding, which affects their target cells (8–10). MVs are fragments of membranes that, in physiological and pathological conditions, evaginate from the membrane surface of normal and tumor cells. MVs vary from 100 to 1000 nm in diameter. The size and content may differ depending on their cells of origin. MVs are able to deliver the contents of the source cell, including proteins, RNA, microRNA (miRNA), and DNA, to the target cells, thereby affecting the target cell and mediating cell-to-cell communication (11, 12). It is known that the leukemic cells interact with normal MSCs in the bone marrow. Several studies have shown the role of MVs in the communication between cancer cells and their environment. These studies have reported that leukemic cell MVs could initiate malignant transformation in normal hematopoietic cells, which could change the phenotype of normal cells into leukemic phenotype (13, 14). However, data concerning the effect of K562-MVs in the hBM-MSCs interactions are missing. We hypothesized that this interaction might be regulated by K562-MVs transferred to hBM-MSCs, governing tumor development and leukemic progression. According to the pathogenesis of leukemia that is stringently associated with a tumor-supportive microenvironment, leukemia-derived MVs contribute to the cellular and molecular mechanisms by which malignant cells create this favorable surrounding (15). Therefore, we hypothesized that K562-MVs might be able to change the phenotype of normal hBM-MSCs to a leukemic-like phenotype, enhance proliferation and increase resistance to apoptosis. In this study, we used hBM-MSCs as a target for MVs derived from the K562 cell line, representative of CML, to seek evidence of apoptosis or survival of hBM-MSCs *in vitro*.

## Materials and Methods

***K562 cells culture***

The Human CML cell line, K562, was obtained from the National Cell Bank of Iran, Pasteur Institute of Iran. The viability of the cells was detected by trypan blue staining (Sigma-Aldrich, USA). Then, the cells were cultured in RPMI 1640 medium (GIBCO, USA). This medium was supplemented with 10% fetal bovine serum (FBS) (Gibco, USA). Cells were grown at 37 °C with a 5% humidified CO_2 _incubator (16). Cultured cells were centrifuged at 300 g at 25 °C for 5 min then the supernatant was collected and stored at -20 °C for MVs isolation.

***Isolation of MVs***

For MVs isolation, cell-free supernatant was obtained from the previous stage and centrifuged at 2,000 rpm at 4 °C for 10 min. Then, to remove all debris and apoptotic bodies, the supernatant was centrifuged at 7000 g and 4 °C for 20 min. The remaining supernatant was ultracentrifuged at 20000 g for 1 hr at 4°C twice to obtain MVs. The protein concentration of isolated MVs was measured with the BCA Protein Rating Kit (Thermo Fisher Scientific, Fisher Scientific, USA) in accordance with the manufacturer’s instructions (17).

***Dynamic light scattering technique (DLS)***

The DLS technique was used to measure the diameter of MVs. In this method, the absorption of MVs suspension in 1 ml PBS was measured at 630 nm by Zetasizer set (ZetaSizer Nano ZS, ZEN 3600, Malvern Company, UK) (17).

***Transmission electronic microscope (TEM)***

The morphology of the MVs was visualized using TEM (Philips, CM20) to confirm that we had isolated MVs. Isolated MVs were stained with 2% -uranyl acetate on formvar-carbon-coated grids as a negative stain. In order to provide transmission images, the grid was placed in the electron microscope after drying (17).

***Human bone marrow-derived MSC (BMSC) culture***

Human BMSCs were obtained from Stem Cell Technology Research Center (Tehran, IRAN) and were cultured in Dulbecco’s modiﬁed Eagle’s medium (DMEM) supplemented with 10% FBS (Gibco, USA) and 1% penicillin-streptomycin (Gibco) in a humidified incubator in 5% CO_2_ at 37 °C. After incubation for 48 hr, the medium was changed, and almost all the non-adherent cells were washed away. Afterward, the medium was changed every 3 or four days. When the monolayer of adherent cells reached 80% confluence, they were isolated by 0.25% trypsin-EDTA (Gibco, USA) and passaged (17). Cells were seeded in 6-well tissue culture plates and then treated with isolated MVs at 0, 15, 30, and 60 µg/ml. Cells in the control group were not treated.

***Detection of MVs in hBM-MSCs***

The fluorescence microscope (Eclipse-TE2000-E, Japan) was used to track and detect the fusion of MVs with the target cells. One ml of MVhhrhs in phosphate-buffered saline was labeled with 1 µl CFSE (Invitrogen, USA) solution before being added to 5 ml DMEM with 10% FBS and incubated in the dark for 15 min (37 °C, 5 % CO_2_). Then, it was centrifuged at 20000 g at 4 °C for 60 min to remove the extra CFSE and to provide the pellet of MVs. Finally, MVs were resuspended in DMEM and added to hBM-MSCs in DMEM with 10% FBS and incubated for 60 min (37 °C, 5 % CO_2_). A fluorescence microscope was used to provide images (17). 

***Microculture tetrazolium test (MTT assay)***

The effect of different concentrations of MVs on the viability of hBM-MSCs was assessed by MTT assay. hBM-MSCs (5,000 cells/100 µl/well) were plated onto 96-well plates and treated with different concentrations of MVs at 0, 15, 30, and 60 µg/ml. After 48 hr, the cells were further incubated with 10 µM of MTT (Sigma, Chemical, St Louis, MO, USA) solution at 37 oC for 4 hr. Untreated cells were used as control. The reaction was stopped by adding 10 µM dimethyl sulfoxide (DMSO). A microplate reader (Anthos 2020, Austria) was used at 570 nm to assess the optical density (17, 18).

***Annexin V FITC assay***

After treatment, apoptosis was detected by flow cytometry using AnnexinV FITC/Propidium iodide (PI) apoptosis detection kit (eBioscience, USA). The cells were washed in cold PBS and resuspended in an Annexin-binding buffer containing Annexin V FITC and PI. The cells were then incubated at room temperature for 15 min in the dark and analyzed by BD FACScalibur flow cytometry. Viable cells are not reactive to AnnexinV and PI (17, 19).

***Analysis of gene expression by real-time quantitative PCR***

Trizol (Invitrogen, USA) was used for extracting total RNA from the cultured cells at days 0, 3, and 7 post-treatment according to the manufacturer’s protocol. Then, cDNA was synthesized using PrimeScript 1st strand cDNA Synthesis Kit (Takara, JAPAN) following the instructions of the manufacturer. Changes in mRNA expression of *KI67* as a cell proliferation marker, *BCL-2* as a prosurvival gene, *BAX *as a pro-apoptotic gene, and *ACTB* as a housekeeping gene were surveyed by a real-time PCR detection system (StepOne Real-Time PCR System, Applied Biosystems, USA) using the SYBR Green master mix (Takara, Japan) according to the manufacturer’s protocol. Fold-change gene expression in MVs treated cells with respect to the control cell line was calculated for all samples based on the 2^(^^-^^∆∆Ct) ^ formula (17, 20).

Primers sequences used are as follows:

Forward: 5’-GTACTTAAAAAATACAACATCACAG-3’ and

Reverse: 5’-CTTGATTCTGGTGTTTCCC-3’ for *BCL-2*;

Forward: 5’-CAAACTGGTGCTCAAGGC-3’ and

Reverse: 5’-CACAAAGATGGTCACCGTC-3’ for *BAX*;

Forward: 5’-TTCTGACCCTGATGAGAGTGAG-3’ and

Reverse: 5’-GAGAGGCGTATTAGGAGGCA-3’ for *KI67*; and Forward: 5’-TGAAGATCAAGATCATTGCTCCC-3’ and Reverse: 5’-AGTCATAGTCCGCCTAGAAGC-3’ for *ACTB*.

***Alizarin Red staining***

The differentiation of hBM-MSCs to osteoblast cells was investigated using Alizarin Red staining (21). Following 14 days of hBM-MSCs culture, the medium was removed and washed twice with PBS. The cells were fixed with 95% ethanol for 10 min and washed twice with PBS. Finally, Alizarin Red 1% (Sigma, USA) was added for 10 min and then washed three times with PBS.

***Oil red O staining***

Oil red O staining was used to detect the differentiation of hBM-MSCs to adipocyte cells (21). Following 14 days of hBM-MSCs culturing, the medium was removed and washed twice with PBS. The cells were frozen at -20˚C for 20 min, then fixed with 4% paraformaldehyde (PFA) for 20 min and washed twice with PBS. Then, 1 ml of 60% isopropanol and 0.5% Oil red O solution (Sigma) were added to the flask for 20 min, and the cells were subsequently washed twice with PBS.

***Statistical analysis***

All data were presented as mean ± SD and were performed in triplicate. GraphPad Prism Software (version 7.00 for Windows, GraphPad Software, La Jolla, California, USA) was used for statistical analysis. The statistical analysis was cried out by Two-Way ANOVA. *P*<0.05 was taken as significant.

## Results

***Morphology of K562 cells***

Human CML cell lines K562 were cultured, and since more than 95% of the cells were viable, their supernatant was collected for MVs isolation. Cultured K562 cells are non-adherent and rounded.

***Detection of the diameter of MVs***

**Figure 1:**
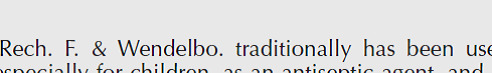
(A) The results of dynamic light scattering technique (DLS) that confirmed the diameter of isolated microvesicle (MV). (B) Transmission electron microscopy image of isolated microvesicles

The diameter of the isolated MVs was measured using the DLS technique. Particles had an average diameter of 237 nm (Figure 1A). This result confirmed that the correct protocol was used for isolation, and the diameter of isolated MVs was in the normal range of 100-1000 nm.

***Transmission electron microscopy***

As shown in Figure 1B, the membrane and the structure of MVs were not damaged during isolation. We used this method to find that the membranes of the MVs completely retained their quality during the process of isolation, indicating the correct protocol for isolation. Also, the size of MVs on electron microscopy was qualitatively confirmed to be less than 200 nm, which is in the size range of 100–1000 nm.

***Detection of MVs in hBM-MSCs***

**Figure 2:**
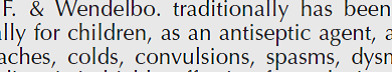
Microscopic image of detecting microvesicles labeled with CFSE solution (X10 magnification), the right one was provided with an optical microscope. The left one was provided with a fluorescence microscope

Detecting the ability of MVs to cross a membrane of hBM-MSC was followed by treatment of hBM-MSCs with MVs labeled with CFSE solution. The entrance of MVs to the target cells was observed with a fluorescence microscope (Figure 2).

***The effect of different doses of MVs on hBM-MSCs***

**Figure 3:**
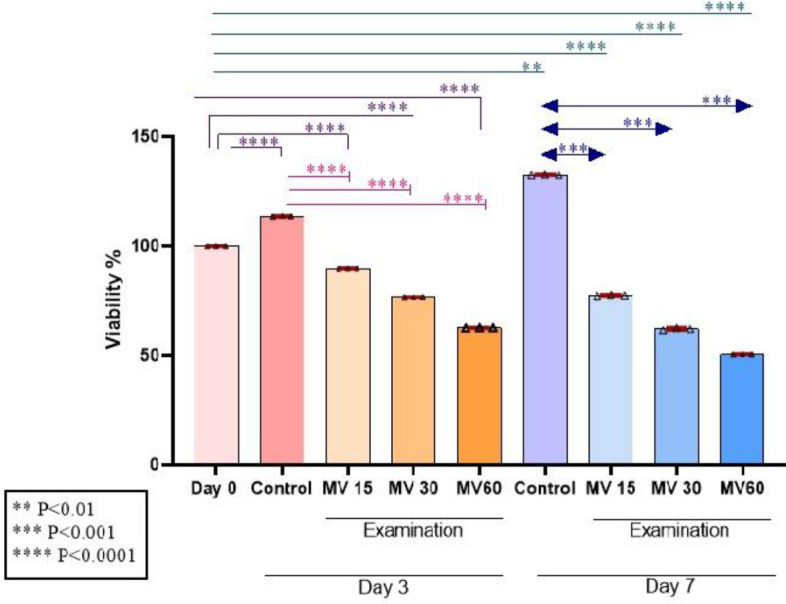
Investigation of cell survival by MTT on days 0, 3, and 7 at different doses of microvesicle treatment. Results showed the dose-dependent effect of MVs on reducing the viability of hBM-MSC

MTT: Microculture tetrazolium test; MVs: Microvesicles; hBM-MSC: Human bone marrow mesenchymal stem cells

The viability of hBM-MSCs was assessed in the non-treated group (as control) and in the treated groups with different doses of MVs on days 0, 3, and 7. As shown in Figure 3, by increasing the dose of MVs, the viability of the cells was significantly reduced compared to the control (dose-dependent). The mean viability between the control and treated groups was significant (*P*<0.05).

***Effects of K562-MVs on apoptosis in hBM-MSCs***

**Figure 4.**
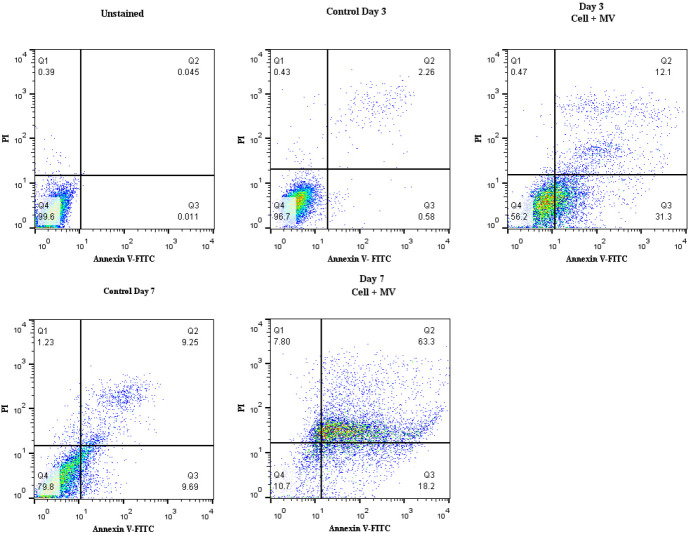
Apoptosis analysis. These diagrams demonstrate the apoptosis of hBM-MSC treated with 30 µg/ml k562-MVs by annexin-V/PI staining for the indicated times. Upper and lower right quadrants were used to measure apoptotic cells. The control has been used to show hBM-MSC without any MV treatment. Live cells are negative to annexin-V and PI; early apoptotic cells are positive to annexin-V and negative to PI, late apoptotic and necrotic cells are positive to annexin-V and PI

Annexin V/PI staining was used to detect apoptosis of hBM-MSCs treated by K562-MVs through flow cytometry. The results showed an increase in apoptotic cells compared to the controls, which demonstrated that K562-MVs treatment with hBM-MSCs at the dose of 30 µg/ml MV for 3 and 7 days induced apoptosis in hBM-MSCs. As shown in Figure 4, the apoptotic effects of K562-MVs on day 7 were more than on day 3. These data showed that K562-MVs induced apoptosis in hBM-MSCs. These data contradict our primary hypothesis that K562-MVs could govern tumor development by changing the phenotype of normal hBM-MSCs to enhance proliferation and contribute to a tumor-supportive microenvironment.

***Differentiation into osteocytes and adipocytes***

**Figure 5:**
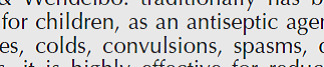
Oil red O and Alizarin Red staining on day 14. (A) Oil red O staining of hBM-MSC exhibit no adipogenic differentiation in the presence or absence of MVs during 14 days. (B) Alizarin Red staining of hBM-MSC exhibited no osteogenic differentiation in the presence or absence of MVs during 14 days

On day 14 of the hBM-MSCs culture, adipogenic and osteogenic differentiation in the presence and absence of MVs was assessed by Oil red O and Alizarin Red staining, respectively. Results exhibited no differentiation in all groups (Figure 5). It shows that MVs did not affect the differentiation of hBM-MSCs during 14 days. 

***hBMSC-MVs decreased BCL-2 and KI67 and increased BAX gene expression***

**Figure 6:**
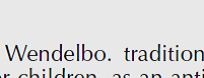
(A) *BCL-2 *gene expression in the studied groups on days 0, 3, and 7. (B) *KI67* gene expression in the studied groups on days 0, 3, and 7. (C) *BAX* gene expression in the studied groups on days 0, 3, and 7

Quantitative real-time PCR showed decreased *BCL-2 *and *KI67* gene expression in hBM-MSCs treated with 30 µg/ml MVs. In this study, the expression of *BCL-2 *and *KI67* decreased by 0.6% and 0.16% on day 3 and by 0.1% and 0.1% on day 7, respectively (Figure 6A and 6B). As shown in Figure 8C, a significant increase in expression was observed in different experimental groups compared to the controls and day 0. *BAX* expression increased by 2.6% on day 3 and 5.7% on day 7 after MVs treatment. 

CFSE: Carboxyfluorescein diacetate, succinimidyl ester

hBM-MSC: Human bone marrow mesenchymal stem cells; MVs: Microvesicles; PI: Propidium iodide

hBM-MSC: Human bone marrow mesenchymal stem cells; MVs: Microvesicles

## Discussion

In recent years, the importance of tumor microenvironment for cancer progression is becoming widely recognized. MSC-based therapy has emerged as a promising therapeutic strategy for several malignancies. MSCs are multipotent cells in the bone marrow stroma, providing a supportive microenvironment for the differentiation of HSCs into functional adult cells hence playing an important role in the pathogenesis of hematological disorders. In order to provide an appropriate microenvironment, various cells in the bone marrow should interact with each other (6, 7). There are several ways for cells to communicate, such as the secretion of cytokines and cell-cell interaction. In this way, Fathi *et al*. in 2019 showed that the co-culture of BMSCs with K562 cells could secrete a substantial amount of TIMP-1 and CINC-1. These cytokines could inhibit the K562 cell proliferation via BAX and caspase-3 cascade pathways (20). So the communication between cells in the bone marrow microenvironment may affect leukemic progression. As shown in several studies, cells may exchange their information by secretion of MVs (22, 17). These membrane-bound vesicles containing bioactive factors play a pivotal role in cell-to-cell interactions. They can control the target cell processes via the transfer of several biomolecules, such as miRNAs, and depending on their cell origin, MVs may be able to reprogram the behavior of the target cells (23–25). According to this point, MVs can be considered as a way cells interact with each other directly by activating cell surface receptors via protein and lipid ligands or by integrating their membrane content into the plasma membrane of their cellular targets (17, 26). The importance of this interaction becomes more highlighted when one of the cells is leukemic, and the other is normal. In this case, studying the effects of this interaction can help better understand the behavior of normal and leukemic cells in the bone marrow microenvironment. 

This study is the first investigation seeking the effect of K562 leukemic cell MVs on the hBM-MSCs, the most important cells in the bone marrow microenvironment. MVs participate in many physiological events and play a key role in regulating programmed cell death. Zhao *et al*. in 2017 showed the apoptotic effect of multiple myeloma-MVs (MM-MVs) in the human kidney-2 (HK-2) cell line. They found that MM-MVs significantly inhibited viability and induced apoptosis through the increase of caspases 3, 8, 9, and E-cadherin. Their results confirmed that Bcl-2 family members were involved, with the upregulated expression of pro-apoptotic regulators Bim and Bid and the downregulated expression of anti-apoptotic regulators Bcl-xl and Bcl-2 (27). On the other hand, Paggetti *et al*. demonstrated that chronic lymphocytic leukemia microvesicles (CLL-MVs) induced MSCs to mimic the phenotype of cancer-associated fibroblasts (CAFs). In this way, tumor cell MVs created a favorable environment for promoting CLL progression. They showed that CLL-derived MVs were actively incorporated by endothelial and MSCs *ex vivo* and *in vivo*. Furthermore, the transfer of exosomal proteins and miRNAs induced an inflammatory phenotype in the target cells, which resembles the phenotype of CAFs. As a result, stromal cells showed enhanced proliferation, migration, and secretion of inflammatory cytokines, contributing to a tumor-supportive microenvironment (15). Also, it has been demonstrated that MVs circulating in the plasma of B-cell chronic lymphocytic leukemia (CLL) patients can activate the AKT/mammalian target of rapamycin/p70S6K/hypoxia-inducible factor-1α axis in CLL BMSCs with the production of vascular endothelial growth factor, a survival factor for CLL B cells (28). However, we demonstrated the apoptotic effect of K562-MVs on target cells. Our results indicated that the expression levels of *KI67* and *BCL-2* as representatives of cell proliferation and survival markers (29, 30) decreased; on the other hand, the *BAX* expression levels increased. Besides, we observed more increments and decrements in the MVs-treated group on day 7 compared to the same treated group on day 3. Also, the difference between treated and control groups in all individual experiments was statistically significant (*P*<0.05). These results were much more remarkable when we observed the analysis of the results of annexin V/PI staining flowcytometric diagrams on day 3 and day 7, which also revealed the apoptotic effect of these MVs in hBM-MSCs, confirming our gene expression results. Also, during these changes, no differentiation into the osteocytes and adipocytes was seen in MSCs after day 14 of the experiment; thus, we may conclude that this treatment is not able to induce differentiation in hBM-MSCs. 

It should be pointed out that our results contrast with some studies which demonstrated the anti-apoptotic effect of tumor cell MVs on the target cells. For example, Zhu *et al*. in 2014, showed the anti-apoptotic effect of K562 leukemia cell MVs on mononuclear cells. They showed decreased *P53* gene expression in mononuclear cells treated with K562-MVs. They found that MVs derived from K562 leukemia cells contained the breakpoint cluster region-Abelson leukemia gene human homolog 1 (*BCR-ABL1*) mRNA. Following incubation with BCR-ABL1-positive MVs, they showed mononuclear cells derived from normal transplants exhibited a leukemia-like malignant phenotype both *in vitro *and *in vivo*. They also demonstrated that MVs contributed to genomic instability by two distinct pathways: via consequent overexpression of activation-induced cytidine deaminase and reactive oxygen species, which mediated DNA breakage and recombination, and via upregulation of methyltransferases and global DNA hypermethylation (13).

Taken together, the ability of leukemia stem cells in the transcriptional programming of the normal MSCs is important in supporting leukemogenesis and tumor progression (31). As Kim *et al*. demonstrated, leukemia stem cells induce a change in the transcriptional programming of the normal MSCs. The modified leukemic niche alters the expressions of cross-talk molecules (i.e., CXCL12 and JAG1) in MSCs to provide a distinct cross-talk between normal and leukemia cells, selectively suppressing normal primitive hematopoietic cells while supporting leukemogenesis and chemoresistance (31). Thus, the remodeling of the mesenchymal niche by leukemia cells is an intrinsic self-reinforcing process of leukemogenesis. Here, we designed our experiment based on the role of tumor MVs to seek their ability to reprogram the normal MSCs. MSCs affected by leukemic MVs went into the apoptosis phase slowly, and *BAX* expression, a promoting apoptosis protein (32), increased after seven days. So we demonstrated that MVs derived from the K562 cell line could induce cell death and apoptosis in hBM-MSCs; furthermore, K562-derived MVs could affect the expression levels of *KI67*, *BCL-2*, and *BAX* genes in all studied groups confirming the molecular mechanisms of the cell death induction in hBM-MSCs *in vitro*. 

## Conclusion

The interaction of leukemic cell-MVs with hBM-MSCs in the bone marrow microenvironment is essential to realize possible pathways of leukemia progression. In this study, we focused on the apoptotic effect of K562-MVs on hBM-MSCs. Still, the exact mechanisms of MV-mediated cross-talk between cells are currently lacking, so further studies are needed to clarify the molecular mechanisms involved in leukemia regulation by leukemic-derived MVs. In this context, the modulation of the function of tumor-MVs may be proposed as a tool for cancer therapy. These findings presented a better understanding of the changes made by K562-MVs to normal hBM-MSCs. However, animal models are also needed to be investigated further.

## Authors’ Contributions

MHM, MS and AA designed the experiments; NR and MAD performed experiments and collected data; MAF, AA and SS discussed the results and strategy; MHM supervised, directed and managed the study; NR, MAD, MS, AA, MHM, MAF and SS final approved of the version to be published 

## Funding

This research was supported in part by Shahid Beheshti University of Medical Sciences, Tehran, Iran.

## Conflicts of Interest

The authors declare that there is no conflict of interest regarding the publication of this article.
